# Traumatic Epidural and Subdural Hematoma: Epidemiology, Outcome, and Dating

**DOI:** 10.3390/medicina57020125

**Published:** 2021-02-01

**Authors:** Mariarosaria Aromatario, Alessandra Torsello, Stefano D’Errico, Giuseppe Bertozzi, Francesco Sessa, Luigi Cipolloni, Benedetta Baldari

**Affiliations:** 1 Legal Medicine Division, Sant’Andrea University Hospital, 00189 Rome, Italy; aromatario@hotmail.com; 2 Section of Legal Medicine, Department of Clinical and Experimental Medicine, University of Foggia, Ospedale Colonnello D’Avanzo, Via degli Aviatori 1, 71100 Foggia, Italy; alessandra.torsello@uniroma1.it (A.T.); francesco.sessa@unifg.it (F.S.); luigi.cipolloni@unifg.it (L.C.); 3 Department of Medical, Surgical and Health Sciences, University of Trieste, 34100 Trieste, Italy; stefanoderrico@hotmail.com; 4 Department of Anatomical, Histological, Forensic and Orthopedic Sciences, Sapienza University of Rome, 00186 Rome, Italy; benedetta.baldari@uniroma1.it

**Keywords:** epidural hematoma, subdural hematoma, interdural hematoma, forensic histopathology

## Abstract

Epidural hematomas (EDHs) and subdural hematomas (SDHs), or so-called extra-axial bleedings, are common clinical entities after a traumatic brain injury (TBI). A forensic pathologist often analyzes cases of traumatic EDHs or SDHs due to road accidents, suicides, homicides, assaults, domestic or on-the-job accidents, and even in a medical responsibility scenario. The aim of this review is to give an overview of the published data in the medical literature, useful to forensic pathologists. We mainly focused on the data from the last 15 years, and considered the most updated protocols and diagnostic-therapeutic tools. This study reviews the epidemiology, outcome, and dating of extra-axial hematomas in the adult population; studies on the controversial interdural hematoma are also included.

## 1. Introduction

Epidural hematomas (EDHs) and subdural hematomas (SDHs), or so-called extra-axial bleedings, are common clinical entities after a traumatic brain injury (TBI), and both are often present in the same subject [1].

Cases of traumatic EDHs or SDHs analyzed by forensic pathologists include road accidents, suicides, homicides, assaults, domestic, and on-the-job accidents, which are all common scenarios for subjects with extra-axial hematomas, either isolated or associated with multiple traumatic injuries [2]. In addition, these widespread pathological entities pose many problems in the field of medical liability. Often a medical examiner is called to rule whether a subject died as a direct consequence of the trauma, or because of incorrect handling by the hospital, or whether possible comorbidities could have played a role in the cause of death. Finally, the medical examiner may be requested to determine the prognosis after a traumatic extra-axial hematoma and, consequentially, its role in the death of the subject, based on the autoptic results alone. This can be the case, for example, for a subject found dead after an assault with many injuries, among them an intracranial hematoma or, in the case of a victim of a hit-and-run, when it is necessary to establish whether the failure to rescue caused the death, or if the TBI was the cause of death. In the context of medical liability, a typical question concerns the timing of a medical treatment, or if the time between injury and surgical intervention influenced the outcome.

The aim of this review is to provide an overview of the published data in the medical literature, to help medical examiners answer such questions. We mainly focused on the data from the last 15 years and considered the most updated protocols and diagnostic-therapeutic tools. In this study, we review the epidemiology, outcome, and dating of extra-axial hematomas in the adult population; studies on the controversial interdural hematoma are also included.

## 2. Methods

Information sources and search, for this literature review, consisted of searching two different databases (PubMed and Google Scholar). A primary selection was conducted with a search strategy, i.e., ((epidural hematoma) OR (subdural hematoma) OR (extra-axial bleeding)) AND ((forensic medicine) OR (legal medicine) OR (medical jurisprudence)); subsequently, for each paragraph of the planned review, i.e., ((epidural hematoma) OR (subdural hematoma) OR (extra-axial bleeding)) AND (epidemiology). In addition, for paragraph on outcomes, the GCS (Glasgow Coma Scale) and the GOS (Glasgow Outcome Scale) were used as search flags. The majority of the studies were categorized based on outcome according to the GOS, which assigns a number between 5 and 1 relative to whether a patient presents as follows: 5, good recovery; 4, moderate disability; 3, severe disability; 2, vegetative state; 1, death. A score between 5 and 4 is considered to be a favorable outcome; the remaining categories describe unfavorable outcomes [3].

For study selection and the data collection processes, first, the initial literature searches were conducted. The papers were selected after the evaluation of the title and abstract, and then potentially relevant studies were further assessed for eligibility. Full-length articles were preferred; duplicate manuscripts or only-abstract-available texts were excluded.

All studies assessing the phenomenon of EDH and SDH linked to TBI were eligible for review, excluding those in which bleeding and trauma were not present. The inclusion criteria were publication date from 1 January 2005 to 31 December 2020, English language, and published in a scholarly peer-reviewed journal. Studies involving a majority of adult subjects were further selected. Moreover, the references of the selected articles were also reviewed.

Following these procedures, 138 eligible empirical studies were included in the present review.

## 3. Epidemiology

According to the literature, SDHs of a traumatic nature affect men more than women, especially aged <50 years [4,5,6,7,8,9,10]. Similarly, EDHs are more frequent in men [11,12,13].

SDHs are generally associated with high-energy traumas, especially road accidents [4,6,7,8,14,15,16]. In contrast, excluding the case study by Zhang et al. [11], EDHs with a clear prevalence of motor vehicle accidents (MVA), showed overlapping percentages of falls and MVAs [17,18,19].

The studies by Han and Shibahashi [20,21] confirmed that in cases of severe TBI, SDHs are much more frequent than EDHs (5:1 ratio), whereas in the case of mild TBI, this ratio is approximately 3:1.

Among the extra-axial hematomas, EDHs have the most favorable prognosis. In 2015, Bir et al. [22] analyzed a large number (more than 5000) of subjects hospitalized in the USA, from 2003 to 2010, with an EDH diagnosis. Approximately half of the subjects were under 18 years of age, and the authors reported a mortality rate of 3.6%. Moreover, the research performed by Ruff’s English and Welsh team [23], in 2012, on another large cohort found mortality rates of 2 and 3% (between surgically treated and not, respectively). These results are in line with studies on fewer cases, in which the mortality rate for EDHs varied between 5.6 and 10% [17,18].

SDHs have a perioperative mortality rate (death occurring within 30 days after surgery) between 11.5 and 67.1% (mean 38.4%, median 38.2%) [4,5,6,7,8,9,10,14,15,16,24,25,26,27,28,29,30,31,32,33,34,35,36,37,38,39,40,41,42,43,44,45,46,47]. This wide range is due to the great diversity of the analyzed cohorts; among them, only two studies [41,42] narrowed the mortality between 14% and 33.5%, respectively. In particular, according to Ryan et al. [41], only a minority (17%) of cases needed surgery for the hematoma following a mild cranial trauma. Taking these observations into consideration, a case study of only SDHs associated with mild TBI reported a mortality of 4% [48].

Conversely, the higher mortality rates reported come from analyses of patients with SDHs associated with more severe TBI [4,5,8,25,28,34,35,36,37,38]. In fact, SDHs with minimal trauma or not immediately treated surgically are generally considered as a stand-alone entity [49,50,51], and they are generally excluded from published cohorts, which partially explains the difference in mortality rates between the two kinds of hematoma.

Posterior fossa subdural hematoma (PFSDH) has a more severe prognosis, as the posterior fossa involves delicate structures for vital functions, and these have their own dedicated studies [52,53]. Similar considerations were made for posterior fossa epidural hematomas (PFEDH), which are associated with a worse prognosis than “common” EDHs (3.4 and 11%, mean 5.5%, median 4.6) [54,55,56,57,58,59,60]. In contrast to the literature, Shibahashi et al. [61] reported a mortality rate of 16.9% and that this kind of hematoma was more frequent in the case of mild TBI.

A more complex topic is whether the elderly are a subgroup with specific mortality rates. In the case of head trauma, the question is even more relevant: elderly people often use antiplatelets and/or anticoagulants, they are at higher risk of falls [62], they often present multiple comorbidities (including homeopathies and coagulopathies that cause hemorrhagic diathesis) [63,64], they have frailer blood vessels [65], and they have a lessened capacity to recover their neurological functions [66]. These characteristics easily indicate that the elderly are more at risk of intracranial hemorrhage, and have a worse prognosis, than the rest of the adult population. There are nine studies that have focused on subjects more than 65 years with SDHs [24,26,30,32,33,40,43,46,67], whereas only two studies focused on EDHs [68,69], and one study [70] analyzed both SDHs and EDHs.

In the adult population accessing an Emergency Department (ED) for a traumatic brain injury, more cases of SDHs than EDHs are shown in elderly patients [70,71,72,73]. Unlike the general adult population, the percentage of elderly men is comparable to that of women [24,26,32,33,40,43,46] in the case of both SDHs and EDHs. In particular, elderly patients affected by an EDH are a minority of the patients accessing an ED (3%) [68]. Moreover, the most frequent causes were fall at home or assault, with different mortality rates (7.1 vs. 25%, respectively) [68,69]. The percentages of associated cranial fractures (57% and 63%) and the most common positioning of the hematoma (parietal) are similar.

SDHs cases in the elderly are most often the result of falls, especially from low heights and in a domestic environment [74,75]. Traffic accidents are the cause in a minority of cases as along with assault and head blunt trauma [24,32,40,43,67,71]. Teo et al. [74] gathered data on the dynamics of falls, showing how SDHs in the elderly are caused by backwards falls in 43.8% of cases, by sideways falls in 31.3% of cases, and at home in 74.1% of cases. This last number contrasts with the data published by Petridis et al. [40], which state that only 38% of cases are due to falls in a domestic environment. Taussky et al. [43] analyzed the laterality of SDHs in the elderly, and they did not find any clear prevalence of laterality nor any impact on the prognosis. Laterality and mortality/favorable outcome do not seem to be correlated, even in the general population. Hsieh et al. [32] analyzed the differences between a cohort of elderly patients and one of adults, and they did not find any significant differences in GCS at admission, hematoma volume at CT examination, and indication to surgically remove the hematoma between the two populations. Lenzi et al. [36], instead, found a higher percentage of surgical decompression among the elderly population.

Studies on EDHs often review data relative to cranial fractures, with percentages between 57 and 87% (mean 69%, median 67.5%) [17,18,19,68,69,76]. In cases of PFEDH, the percentage of fractures is even higher, variable between 50 and 100% (mean 87.3%, median 93%) [54,55,56,57,58,59,60]. Conversely, studies on SDHs rarely report data on the presence of concurrent cranial fractures. Kuhn et al. [33] reported 7.6% of SDH cases were associated with a cranial fracture in the elderly; Alagoz et al. [14] reported the percentage in the general population as 26.3%. With so little data, it is not possible to offer significant hypotheses on this difference; however, it could probably be due to the prevalence of road accidents in falls in the respective cohorts. Sawauchi et al. [42] instead observed how patients with SDH and more severe head trauma presented fractures at a much higher percentage when compared to patients with SDH without signs of cerebral edema (62.3 vs. 27%). Thus, contrary to EDHs, SDH shows a more significant relationship between the presence of fractures and severity of trauma. When we look at studies on maxillofacial fractures associated with SDHs, we can see how this kind of lesion is caused by blunt trauma, especially in assaults, and less frequently in MVAs. Generally, when the SDH is associated with a maxillofacial fracture, trauma is more severe. Moreover, often these fractures are in the mid-face area (the worst of the maxillofacial fractures) and are rarely in the low face area. EDHs are much less frequently associated with maxillofacial fractures and, generally, the mid-face area [77,78].

According to the data, EDHs have favorable outcome—that is to say a GOS at discharge/follow up of 4 or 5 (good recovery and moderate disability)—between 69 and 95% (mean 84.3%, median 88.9%) [11,12,17,18,19,76,79], whereas the range of SDHs varies between 9 and 76% in the perioperative period (mean 32.1%, median 26.5%) [4,5,6,8,15,16,24,25,26,28,29,33,36,37,39,40,41,42,43,44,46]. An increase in these percentages is revealed during follow up (variable, according to the single case, from 3 months to 1 year). This increase is mostly due to the functionality recovery of patients with GOS 3 [6,24,26,46]. Generally, the highest percentages of functionality recovery are found in studies that report only follow up data, without the perioperative data [47,80].

As stated before, studies on elderly patients with EDHs are difficult to compare. If we instead compare the specific cohorts of elderly patients with SDHs, the perioperative mortality rate varies between 17.6 and 55.1% (mean 36.4, median 35%) [24,26,30,32,33,40,43,46,67]. These results are the same as the ones in the general population, even when we take into account the fact that these case studies are fewer in number and have limited cohorts. Moreover, studies that aimed to compare the mortality rate and/or functional recovery between the general population and the elderly have not found statistically relevant differences in the majority of cases [32].

## 4. Outcome

Nowadays, many scores and algorithms have been established to provide useful evaluation of the prognosis of patients with EDHs or SDHs; however, these scores are designed to be used exclusively in hospitals, and they are based on features often unknown to the medical examiner [40,81,82]. Especially in the case of subjects who died before arriving to an ED, the medical examiner can base the evaluation only on the morphology of the hematoma (thickness, presence of MLS, volume) with evident limitations, when compared to the possible analysis with high-resolution imaging techniques on live subjects [83].

Some recent studies have focused on the factors with the strongest prognostic value concerning the in-hospital mortality rate among patients undergoing surgery with isolated craniocerebral lesions (EDHs and SDHs) [84,85,86]. Despite the most commonly used parameters, including the extent of the injury or the time between trauma and surgery, the factors with the strongest prognostic values were represented by clinical parameters (initial GCS score, respiratory rate, blood glucose, blood saturation, systolic pressure, midline shift, and hematoma type). Clinical factors, some of which overlap with those listed above, were also associated with better outcomes six months after discharge from a nosocomial setting (initial GCS score, respiratory rate, saturation, glycemia, and systolic blood pressure), and an assessment of the prognosis in the pathological forensic field will be complicated if there is no adequate health documentation to support this assessment.

Therefore, in the case of SDHs, statistically relevant factors for the prognosis include age, trauma mechanism, morphology of the hematoma, perioperative GCS score, signs of cerebral edema, and timing between trauma and surgical therapy. Studies on the prognosis and risk factors for unfavorable outcomes in patients with EDH are fewer in number, and generally there is less attention on these issues.

### 4.1. EDH

In EDH cases in the general adult (or mostly adult) population, with different forms of head trauma and variously associated other-than-head traumas, only a minority (from 5.6% to 23.3%) of patients have shown an unfavorable outcome [11,12,17,18,19,76,79].

Cheung et al. [18], in a review dealing with isolated EDH cases in 89 patients between 1 month and 87 years old, found that in almost all of the deceased patients (8 out of 9) the GCS score at the time of hospitalization was less than 8; 4 of them had GCS 3, whereas 6 of them had at least one fixed and dilated pupil. All deceased patients were older than 18. Le Roux et al. [69] also found that in the cases they analyzed, not one of the patients who fell into a coma (GCS less than or equal to 8) had a good outcome. On the matter of fixed and dilated pupils, a systematic review and meta-analysis found that in the case of EDH with fixed pupils, the overall mortality rate was 29.7%, and favorable outcome was 54.3% [87].

Hamlat et al. [68] found comparable outcomes (among patients over 65 years old) to those of the general population; specifically, for these patients the main cause of trauma was syncope with a fall, but the prognosis was good as most cases involved trauma of the parietal area, not the temporal one. Conversely, Le Roux et al. [69] found that 34% of subjects made a good recovery or had a moderate disability.

In the case of mild TBI, the presence of EDHs was not found to be associated with unfavorable outcome [88]. Even among patients with mild TBI who underwent brain surgery, EDH was not a predictor of poor neurological outcome [89]. Moreover, Mejaddam et al. [90] point out how among patients with mild and severe TBI, who also underwent surgery, EDH was associated with a high GOS score. On the other hand, in cases of severe TBI, Leitgeb et al. [34] found that the mortality rate in the intensive care unit, during a 90 day period, was 22.2% for EDH alone. Another review by the same author [35] found that 31% of patients with an EDH died in the hospital, and 13% survived with unfavorable outcome. In studies on patients who underwent evacuation surgery and/or decompressive craniectomy, this cohort was also considered as a subgroup affected by more severe traumas. Among them, a study by Moon et al. [91] found a mortality rate of 7–8%, whereas Otani et al. [92] found that 38.2% had good recovery and 14.7 died.

In a study concerning cases of patients with an EDH with complicated supratentorial herniation, 18.2% presented with post-traumatic massive cerebral infarction, and thus received additional decompressive craniectomy and duroplasty; the majority had a good outcome. Among massive cerebral infarction patients with EDH, better prognosis was observed if decompressive craniectomy was performed sooner [93].

Other studies reviewed more specific cases of patients with EDHs. A study by Lau et al. [94] reviewed the cases of 103 adult patients who underwent surgery for an intracranial hematoma; 24 had an epidural hematoma, and their mortality rate was 16.7%, with a return to baseline of 75%. Other studies take into consideration isolated cases of EDHs, but they merely point out the absence of cases of death, and data on good or poor outcomes are lacking [13].

In the case of epidural hematomas, a typical case of medical liability happens when a patient is initially assigned a conservative treatment and then their condition aggravates later. This kind of situation is called by some authors “progressive epidural hematoma”, that is to say, a hematoma with new onset or that increased in size at the time of a second CT [95]. In this concern, Carlson et al. [96] analyzed the security of keeping subjects with mild TBI under observation, as opposed to transferring them to a neurosurgery facility. This study found that subjects with an EDH rarely worsened (1 out of 19; 5.2%). Another study by Basahm et al. [17], on 125 EDH patients under 16 years old who were treated conservatively, showed that only 11.2% later worsened and underwent surgery. Another study [97] confirmed similar values, around 13% (7 out of 54).

In a study by Chen et al. [95], out of 412 patients between 12 and 86 years of age who were hospitalized with head trauma, 38 (9.2%) developed a progressive epidural hematoma, and 15 of these already showed a small EDH at the moment of hospitalization. The hematoma worsened or appeared between 2 h and 7 days, with a peak between 7 and 24 h. The outcome was unfavorable in 36.8% of cases. In a study by Radulovic et al. [98], the outcome of these delayed EDHs was still favorable in the overall cases, with only one patient who was left with moderate disability. In this study, all patients developed an epidural hematoma underlying a skull fracture; moreover, in all three cases that presented with mild head injury GCS > 12, neurological deterioration preceded the detection of a delayed EDH.

As far as the timing of pre-surgery is concerned, two of the examined studies underline how this is not a deciding factor for the prognosis [79]. Neither trauma mechanisms nor the presence of midline shift seem to have a negative influence on the prognosis; conversely, factors such as age, type of hematoma, GCS at the time of hospitalization, and presence of cerebral herniation do. Analogously, Gutowski et al. [12] found no correlation between time of surgery and outcome in the patients, and that midline shift did not influence the outcome. Moreover, the presence of a hematoma with active bleeding is a highly unfavorable factor. This situation can be evidenced through radiological analysis (extraversion of the contrast medium or swirl sign); if a CT scan was used instead, this kind of hematoma will appear with mixed density. Guo et al. [99] found a favorable outcome in 62% of patients with a swirl sign found through CT scan, vs. 85% of patients without it, and a mortality rate of 24% vs. 6%. Pruthi et al. [100] found a mortality rate of over 20% in patients with mixed-density hematomas, despite early surgical intervention. According to Jin et al. [79], another radiological parameter to find patients with unfavorable prognosis would be the pre-surgery and post-surgery GWR (gray/white matter ratio) value, essentially linked to cerebral edema.

Regarding PFEDH prognosis, Jang et al. [56] found that patients with the quickest deterioration were the ones with diastasis of the lambdoid suture, that is to say, the ones where bleeding originated from the sigmoid sinus. Other places of bleeding were the transverse sinus and the small meningeal vessels of the fractured bone. Yilmazlar reviewed cases of 30 patients with non-arterial, bleeding EDHs in the posterior fossa and found an overall mortality of 20% [101].

In the cases of non-arterial bleeding, Giannakaki et al. [102] reviewed EDH cases with detachment of the transverse sinus in the previous 20 years; they found a total of 11 cases of EDH in adults, with a good outcome (GOS 5) in almost all cases.

Yanagawa et al. [103] reviewed cases of EDHs of the temporal tip, and all were treated conservatively. Fatal cases were caused by a deterioration of the associated intracranial lesion. There was no difference in terms of GOS score between EDHs in the temporal tip and other sites.

### 4.2. SDH

No authors have found any correlation between trauma mechanism and prognosis of SDH [4,7,14,15,28,40,71]. Many studies have failed to identify the following prognostic factors as unfavorable: location, thickness, MLS, and the volume of hematomas—that is to say, all characteristics more closely tied to the hematoma itself [4,26,33,40,71]. Other studies [10,14,24,25,28,32,33] have found correlations. Conversely, indirect signs of primary cerebral edema at admission, such as fixed pupils [4,8,15,24,28,40,43] and lower GCS score [4,9,10,14,15,24,26,28,36,40,46,67], were associated with higher mortality and worst outcome. Therefore, it seems that in the case of SDHs the severity of primary cerebral damage, directly caused by the trauma, is the most important factor influencing the prognosis, not the subsequent cerebral suffering, because of the effect of the clot mass formed in the meantime.

This is reflected in “paradoxical” data that show the worst outcomes for patients with the smallest amount of time between trauma and surgical intervention [6,9,10]. It is universally accepted that “time is brain”, even in the case of intracranial hematomas, and that if the patient undergoes surgery less than 4 h from trauma, the prognosis is better [104]. Therefore, severe trauma with compromised GCS is associated with a quicker and more invasive operation. This explains the worse prognosis of subjects who rapidly undergo decompressive craniectomy.

However, only two of the reviewed studies found correlation between increased timing from TBI and operation and unfavorable outcome [14,28]; others did not find any correlation [7,29].

Regarding anticoagulant/antiplatelet use, a statistically significant correlation between higher mortality rates and poor prognosis was described [32], though most of the studies failed to find any relationship [26,29,33,43,67,71], and Walcott et al. [10] found a “paradoxical” protective effect. This can be explained by the fact that antidotes are often available in the pre-operative stage, and thus hemorrhagic diathesis does not influence the surgery risk. Moreover, as stated before, the volume of the hematoma itself is not a negative factor for prognosis; therefore, more severe bleeding, which influences the hematoma itself, and not the primary parenchymal damage cannot play a significant role. Finally, hemorrhagic diathesis is typical in elderly patients [63,64], logically linked to a greater frequency of SDH following a mild TBI; but on the other hand, mild TBI is by definition associated with minor traumas and is associated with a more favorable prognosis [74].

When looking at studies that analyzed the impact of age on prognosis, there is no consensus between on whether age is an unfavorable prognostic factor; only few studies have found a clear correlation [8,9,10,14,15,29], and the majority of them have not [4,7,24,26,43,71].

No authors found any correlation between gender and outcome [4,7,9,14,33,67,71].

## 5. Dating

Dating an intracranial hematoma is a challenge for forensic scientists, especially in SDH cases. This kind of hematoma, seeing as it is generated from the veins, has a slow onset; it can initially be asymptomatic and cause death in the span of days or months [105]. Moreover, it is also possible that a second trauma causes re-bleeding of an already present hematoma [106]. Finally, SDHs are typically associated with abused infants [107,108,109]. For these reasons, and especially because of possible effects that might have a late onset, pathological microscopy reviews have focused on dating the posttraumatic interval in subdural hematomas.

### Dating of SDHs

Even though this matter has relevant forensic implications, there are only few postmortem studies on the dating of subdural hematomas [110,111,112,113,114,115,116]. As verified by Van Der Bos [110], estimated dating times published in main forensic medicine texts have essentially been based on the first histologic study on the topic, published by Munro and Merritt in 1936 [111]. This study takes into consideration very few samples for the various kinds of post-traumatic intervals (PTIs), with only one pool greater than 10. However, even though almost 100 years have passed, the study by Munro and Merrit describes in a valid and precise manner the natural history of a subdural hematoma, from the first phases of organization to complete re-absorption or, in some cases, calcification.

First, an SDH can be divided into three “compartments” with different physiology: the dura–clot interface, the hemorrhagic clot, and the clot–arachnoid interface; the first phase is when fibrin is deposited at the margins of the clot. After this, the fibroblasts in the dura mater start migrating into the fibrin layer. This is the start of the formation of the neomembrane. On the dural side, the neomembrane is more or less of uniform thickness, and it grows until it reaches the same thickness as the meninx. On the arachnoid side, the neomembrane forms later, seeing as the fibroblasts have to migrate to it from the dura mater and through the clot. Moreover, the neomembrane on this side will not have a thickness comparable to the one on the dura mater’s side, and it will have a much less predictable and regular growth.

The red blood cells (RBCs) present in the clot are intact in the first stages; later, they develop irregular shapes and start to lose color. After this, lysis starts until the RBCs disappear completely. This process is much more visible in the center of the clot than in its periphery. At the same time, we can observe the progressive appearance of white cells, firstly granulocytes and macrophages. During the organization of the clot, we can observe RBC containing-macrophages and hemosiderin-containing macrophages. In the later stages, RBCs disappear completely, and at most we can observe deposits of hemantoin and hemosiderin. At the same time, neoangiogenesis starts.

Finally, after months and years, the number of fibroblasts diminishes too and is replaced by hyalinosis-affected connective tissue. This tissue is similar to the dura mater tissue; it can be recognized by the arrangement of its fibers. A clear difference between dural stratus and arachnoid stratus is also difficult to find.

Studies on dating of SDHs are based on these phenomena, especially on the development timing of each one.

In order to date an SDH correctly, it is necessary to observe all three compartments. Authors agree in suggesting to take a sample at the margins of the clot, even when it is not that easy to find or to take a sample from (even in the case of very fresh hematomas with an abundant liquid component). Ideally, taking a sample from the margin not only allows the surgeon to observe the dural side, the clot, and the internal side, but it is also generally easier to handle; areas with greater thickness can still have a liquid component that can hinder sampling. At any rate, it is fundamental to sample the clot together with the dura. In the case of re-bleeding, it is necessary to take multiple samples in the areas that evidently differ.

The majority of authors used traditional histological techniques, mainly hematoxylin eosin (H&E) [110,111,112,113,114] and Perls staining (Prussian blue) to visualize iron [110,112,113,114,115]. Some authors also used immunohistochemical methods, mainly to better distinguish the various white cells and to locate the development of neoangiogenesis as quickly as possible [110,114,115].

Walter et al. [114] analyzed a sample of 222 subdural hematomas using 10 parameters: RBCs, leukocytes, macrophages, RBC-containing macrophages, hemosiderin-containing macrophages, hematoidin, fibroblast invasion, ingrowth of endothelial cells, collagenization, and development of the neomembrane.

Al Sarraj et al. [115] proposed to re-evaluate the traditional PTI based on macrophagic activity between the dura and the clot. They found MHC class II was markedly expressed in the inner aspect of the dura mater within the initial 24 h after injury, while CD68 was quantitatively expressed within the dura 24–48 h after TBI. If PTI was >10 days, MHC class II appeared widespread in the inner context of the dura.

Van der Bos et al. [110] used H&E, elastic van Gieson, and Perls iron stains; additionally, immunohistochemical stains with CD45, CD68, and CD34 were performed (pan-leucocytic markers, for the monocytes lineage and endothelium, respectively). The sample comprised 64 cases, median age 46. In this study, the authors aimed to improve the evaluation criteria used by Munro and Merritt, after confirming their validity, regardless of the limits discussed before. Specifically, the method outlined by Van der Bos simplifies and clarifies some of the original features of Munro and Merritt’s. Moreover, it adds the use of immunohistochemistry, even though the authors underline that their method can be applied with traditional histology alone. They consider their case pool a “widening” of Munro and Merritt’s.

Rao et al. [113] used H&E, Perls stain, Periodic Acid Schiff, and Masson’s Trichrome; the histomorphological features of the dura and the clot analyzed included red blood cells, polymorphonuclear leukocytes (PMN), macrophages, red blood cell containing macrophages, hemosiderin containing macrophages, fibroblasts, collagen fibers, capillary proliferation, and early membrane formation. They observed that RBC lysis partly began at a PTI of 8 h; the majority of cases with complete lysis of RBCs were found to have a PTI greater than 96 h. They also observed that PMNs were present in all cases, even in the early PTI. However, the count was initially low, then gradually increased with the PTI, and finally decreased at higher PTI. Therefore, this study did not find a statistically significant correlation with any PTI, seeing as a significant presence of PMNs was observed even in early specimens. The authors hypothesize that the number of PMNs observable in SDH is impacted by two components: the first would be tied to the PMNs in the blood vessels at the moment of trauma (a factor that is variable and unpredictable); the other one would be PMNs attracted to the site of the hematoma by the inflammatory stimulus.

Delteil et al. [112] proposed a postmortem dating system specific for subdural hematomas in infants under 3 years. The study was based on approximately 57 subjects with a roughly known post-traumatic interval; it used many morphological criteria relative to the dura mater and the clot. Specifically, the study shows how the formation of neomembranes in infants occurs earlier than in adults. However, the authors underline how they analyzed few cases relative to the interval of 4 to 7 days, and over 1 month.

When confronting the data gathered by various studies, it is clear that the first phenomena—which happen only in the first 12 h—are the initial deposit of fibrin and the migration of various components of the white series in the clot. Between 12 and 24 h, it becomes possible to observe the initial migration of fibroblasts and the phagocytosis of RBCs. We start to see a neomembrane after around 4 days, and it will have a definite appearance after around 10 days. Vessel proliferation is not observed before 5 days.

The few studies on the dating of subdural hematomas all have a common limitation: the heterogeneity of the sample. The formation and development of a clot are physiological phenomena that are influenced by a multitude of individual and pathological factors. It is unknown if a sample taken from the center of the clot is comparable to one taken from its periphery; the necessary size for a representative sample is also unknown. Van der Bos et al. [110] suggest to always annotate the dimensions and volume of sampled SDHs. Specifically, one of the most used parameters for dating is the thickness of the neomembrane, but these data are analyzed relative to the volume of the hematoma. Therefore, if we do not have these other data, they suggest not taking the thickness of the neomembrane into consideration when dating an SDH. The analysis of neovessel caliber can also be problematic, seeing as it is subjective and not standardized.

An indirect method of dating is the toxicological analysis of the hematoma itself. If any toxic substance is present in the body at the time of sampling, the difference in concentration of the substance in the clot relative to the peripheral blood can give greater insight on dating the traumatic event, especially in the case of a hematoma that formed slowly [116].

The reviewed studies show how analyses are limited to the clot and the meninges, and how they are based on histological and immunohistochemical analyses, with no use of molecular techniques. Regarding dating of blood clots and hematomas in other parts of the body (spleen, soft tissues, cerebral contusion) [117,118,119,120], traditional histological and immunohistochemical techniques are generally applied (the latter limited almost exclusively to the identification of various types of cells and/or tissue components). Various studies on the dating of hematomas (subdural, but also from other parts of the body) have been published in the field of image-based diagnostics [121,122], but there are no case studies on images acquired post mortem.

## 6. Interdural Hematoma

Recently, a few articles on the development of hematomas between dural layers have been reported in the literature. This type of hematoma can be classified as “interdural”, “intradural”, or “interlaminar”.

The cause is mainly unknown, even though a minority of reports point out a link to traumas and medical procedures (especially during neurosurgery) [123,124,125]. Some cases are secondary to aneurysm rupture in the clinoid region, but it is debated if they develop in the actual interdural space [126,127,128,129,130]. Finally, another group investigated interdural hematomas in foeti and newborns during pregnancy and labor [126].

In all the articles reviewed [124,125,131,132,133,134,135,136], the presence of blood between dural layers was confirmed by imaging and by surgical and microscopic examinations. Blood was always located in the fronto-temporal convexity region, more often on the right side (78%). Usually an interdural hematoma does not reach a great size (especially if it is subacute or chronic), although in some cases it can have a hemispheric distribution [132]. Interdural hematomas are more common in elderly men around 70 years old. The treatment of choice is surgical evacuation (craniotomy or craniostomy). The outcome has been excellent/good in all the reported cases.

In most cases there was no history of trauma, except for one in which the hematoma developed under a bone fracture [133,134]. Thus, in the majority of cases examined, the source of bleeding was not identified, not even with a CT angiography [135]. These results may be due to the fact that dural layers are poorly vascularized and contain few vessels [133].

In two cases, the interdural hematoma iatrogenically developed in the contralateral convexity after brain surgery (for temporal lobectomy and chronic subdural hematoma evacuation) [124,125].

Macroscopical and histological examination showed common findings in the hematomas such as blood clots in various stages of organization [124,125,131,132,133,134,135,136,137,138]. In one case, an immunohistochemical test was performed, confirming the absence of neoplastic cells [133,134]. In 2017, Genc et al. reported a curious case of chronic interdural hematoma that showed calcification and osseous metaplasia with bone and fatty tissue [136].

However, because of the scarcity of reports, the data obtained are inconclusive.

## 7. Conclusions

EDHs and SDHs are very different in epidemiology, clinical presentation, natural history, and outcome. When compared to EDHs, mortality rates for SDHs are significantly higher, and a good outcome is rarer. This reflects the epidemiological differences between the two kinds of bleeding, with SDHs more frequently associated with high-energy traumas and in multiple places. This also reflects a more frequent incidence of cerebral lesions associated with SDH, such as contusions, lacerations, and diffuse axonal injury; all these cause “primary” cerebral damage, and secondary damage is due to the mass effect of the SDH, with associated reduced blood flow and hypoxia of the adjacent parenchyma.

Moreover, the majority of case studies on SDHs focus only on patients who underwent neurosurgery, whereas studies on EDHs generally gather mixed data on patients that might even be treated conservatively. This might be due to the different natural history of the two pathologies. An EDH grows rapidly, and stabilizes just as rapidly, whereas SDH is a venous bleeding and, as such, is less massive. This leads to dramatic developments that need immediate evacuation or, conversely, causes less threatening developments that, if treated conservatively, become chronic. Chronic SDHs have specific clinical and therapeutic characteristics, and they are generally analyzed in dedicated studies. Chronic EDHs are rare, and in the case of a thin layer that does not need evacuation, the EDH generally proceeds towards resolution and does not become chronic.

Another important difference in case studies on extra-axial hematomas is that the majority of studies on a general sample population with EDHs simply report the percentages and/or the number of patients with favorable or unfavorable outcome; they do not report any data on the GOS scale. More specific data are reported, instead, in studies that analyze subgroups of patients with EDHs, especially those with EDHs in the posterior fossa. Data on SDH outcome are generally more detailed and based on significantly larger cohorts. It is of note that the majority of recently published case studies on EDHs are generally based on small cohorts, with less than 100 subjects.

## Data Availability

No new data were created in this study. All analyzed data were obtained from references listed in the specific paragraph.

## Abbreviations

EDH: epidural hematoma; SDH: subdural hematoma; MLS: midline shift; MVA: motor vehicle accidents; PTI: post-traumatic interval; TBI: traumatic brain injury; RBC: red blood cell; PMN: polymorphonuclear leukocytes; H&E: hematoxylin and eosin; GCS: Glasow coma scale; GOS: Glasgow outcome scale; MHC: major histocompatibility complex.
